# Oseltamivir-Resistant Influenza Viruses A (H1N1) during 2007–2009 Influenza Seasons, Japan

**DOI:** 10.3201/eid1606.091623

**Published:** 2010-06

**Authors:** Makoto Ujike, Kozue Shimabukuro, Kiku Mochizuki, Masatsugu Obuchi, Tsutomu Kageyama, Masayuki Shirakura, Noriko Kishida, Kazuyo Yamashita, Hiroshi Horikawa, Yumiko Kato, Nobuyuki Fujita, Masato Tashiro, Takato Odagiri, the Working Group for Influenza Virus Surveillance in Japan

**Affiliations:** National Institute of Infectious Diseases, Tokyo, Japan (M. Ujike, K. Shimabukuro, K. Mochizuki, M. Obuchi, T. Kageyama, M. Shirakura, N. Kishida, K. Yamashita, M. Tashiro, T. Odagiri); National Institute of Technology and Evaluation, Tokyo (H. Horikawa, Y. Kato, N. Fujita); 1 Members of the Working Group for Influenza Virus Surveillance in Japan are listed at the end of this article.

**Keywords:** Viruses, influenza, oseltamivir, drug resistant, neuraminidase, influenza A (H1N1), respiratory infections, Japan, research

## Abstract

To monitor oseltamivir-resistant influenza viruses A (H1N1) (ORVs) with H275Y in neuraminidase (NA) in Japan during 2 influenza seasons, we analyzed 3,216 clinical samples by NA sequencing and/or NA inhibition assay. The total frequency of ORVs was 2.6% (45/1,734) during the 2007–08 season and 99.7% (1,477/1,482) during the 2008–09 season, indicating a marked increase in ORVs in Japan during 1 influenza season. The NA gene of ORVs in the 2007–08 season fell into 2 distinct lineages by D354G substitution, whereas that of ORVs in the 2008–09 season fell into 1 lineage. NA inhibition assay and M2 sequencing showed that almost all the ORVs were sensitive to zanamivir and amantadine. The hemagglutination inhibition test showed that ORVs were antigenetically similar to the 2008–09 vaccine strain A/Brisbane/59/2007. Our data indicate that the current vaccine or zanamivir and amantadine are effective against recent ORVs, but continuous surveillance remains necessary.

## Abstract 2

Prevalence of these viruses increased during the 2008–09 season.

Influenza A and B viruses are major pathogens that represent a threat to public health with subsequent economic losses worldwide (*1*). Vaccination is the primary method for prevention; antiviral drugs are used mainly for prophylaxis and therapy. Currently, 2 classes of drugs, matrix 2 (M2) blockers and neuraminidase inhibitors (NAIs) are available, but M2 blockers such as amantadine and rimantadine are not commonly used because of the rapid generation of resistance and lack of efficacy against influenza B virus (*2*–*4*). The NAIs zanamivir and oseltamivir are widely used because of effects against influenza A and B viruses and a low frequency of resistance. NAI virus surveillance studies by several groups have demonstrated that <1% of viruses tested show naturally occurring resistance to oseltamivir as of 2007 (*5*–*10*), indicating limited human-to-human transmission of these viruses.

At the beginning of the 2007–08 influenza season, however, detection of a substantially increased number of oseltamivir-resistant influenza viruses A (H1N1) (ORVs) was reported, mainly in countries in Europe where the prevalence varies, with the highest levels in Norway (67%) and France (47%) (*11*–*14*). These viruses showed a specific NA mutation with a histidine-to-tyrosine substitution at the aa 275 position (N1 numbering, H275Y), conferring high-level resistance to oseltamivir. Most of these ORVs were isolated from NAI-untreated patients and retained similar ability of human-to-human transmission to oseltamivir-sensitive influenza viruses A (H1N1) (OSVs) (*10*,*15*). In response to public health concerns about ORVs, the World Health Organization (WHO) directed Global Influenza Surveillance Network laboratories to intensify NAI surveillance and announced regularly updated summaries of ORV data collected from each laboratory on its website (*16*). This site reported that the global frequency increased from 16% (October 2007–March 2008) to 44% (April 2008–September 2008) to 95% (October 2008–January 2009), indicating that ORVs have spread rapidly around the world.

Japan has the highest annual level of oseltamivir usage per capita in the world, comprising >70% of world consumption (*10*). Such high use of oseltamivir has raised concerns about emergence of OSVs with increased resistance to this drug. Moreover, in Japan, 2 recent influenza seasons were dominated by influenza viruses A (H1N1) (Figure 1). If a high prevalence of ORVs is observed, primary selection of oseltamivir treatment for influenza patients should be reconsidered. Thus, monitoring ORVs is a serious public health issue.

**Figure 1:**
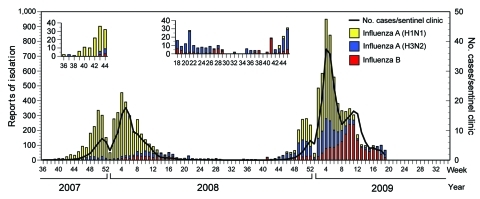
Weekly cases of influenza and isolation of influenza viruses in the 2007–08 and 2008–09 seasons in Japan (as of July 2, 2009). The National Epidemiologic Surveillance of Infectious Diseases (NESID) Network comprises the Ministry of Health, Labor and Welfare; the National Institute of Infectious Diseases; 76 local public health laboratories; ≈3,000 pediatric clinics; and 2,000 internal medical clinics. The NESID Network monitored influenza activity during the 2007–08 season (week 36, September 2007–week 35, August 2008) and 2008–09 season (week 36, September 2008–week 22, May 2009). Clinically diagnosed influenza-like cases were reported weekly by influenza sentinel clinics. **Boldface** line indicates weekly cases of influenza-like illness per influenza sentinel clinic (values shown in right bar). Bars indicate numbers of influenza A (H1N1) (yellow), A (H3N2) (blue), and B (red) isolates (values shown in left bar). Influenza activity started week 47 of 2007 and finished in week 14 of 2008 in the 2007–08 season and started week 49 of 2008 and finished in week 22 of 2009 in the 2008–09 season. Among all influenza isolates, influenza A (H1N1) consisted of 81% during 2007–08 and 49% during 2008–09. Seasonal influenza surveillance showed that influenza viruses A (H1N1) dominated the 2 recent influenza seasons in Japan.

To estimate the frequency of ORVs and characterize these viruses, we analyzed 1,734 clinical samples isolated from the 2007–08 season and 1,482 isolates from the 2008–09 season by NA sequencing and/or NAI inhibition assay. The total frequencies were 2.6% in the 2007–08 season and 99.7% in the 2008–09 season, indicating that ORVs increased dramatically in Japan.

## Materials and Methods

### Virus Testing

Influenza sentinel clinics send clinical specimens to local public health laboratories for virus isolation. Several culture tissues, including MDCK, Caco-2, and LLC-MK2, are used for virus isolation. Without successful viral isolation, clinical specimens are analyzed directly. Influenza viruses were collected from all 47 prefectures in Japan for this study; 1,734 samples of influenza A (H1N1) were isolated during the 2007–08 season (September 2007–August 2008**)** and 1,482 samples of influenza A (H1N1) were isolated in the 2008–09 season (September 2008–April 2009). During the 2007–08 season, viruses were isolated primarily after December 2007. All influenza viruses A (H1N1) were subjected to full or partial (nt 615–1076) NA sequencing to detect H275Y substitution on the N1 NA protein. Representative influenza viruses A (H1N1), including ORVs and OSVs, were subjected to NA inhibition assay (number of tested viruses isolated during the 2007–08 and 2008–09 seasons was 306 and 58, respectively), full NA sequencing (891 and 83), hemagglutinin (HA) 1 sequencing (299 and 83), M2 sequencing (288 and 79), and hemagglutinin inhibition (HI) test (187 and 59).

### Sequence Analysis

The phylogenetic tree of NA and HA1 genes was constructed by neighbor-joining methods. The phylogenetic tree was described by representative ORVs and OSVs isolated from several prefectures in Japan. Sequence information for isolates from other countries was obtained from the Global Initiative on Sharing Avian Influenza Data and the Los Alamos National Laboratory database. All amino acid positions in the phylogenetic tree were described by N1 numbering.

### NA Inhibition Assay

The chemiluminescent NA inhibition assay was performed by using the NA Star Kit (Applied Biosystems, Tokyo, Japan) with slight modifications of the instructions provided by the manufacturer. The final drug concentration ranged from 0.03 nmol/L to 6,500 nmol/L for oseltamivir and from 0.03 nmol/L to 12,500 nmol/L for zanamivir. Chemiluminescent light emission was measured by using an LB940 plate reader (Berthhold Technologies, Bad Wildbad, Germany). Drug concentrations required to inhibit NA activity by 50% (IC_50_) were calculated by a 4-parameter method using MikroWin 2000 version 4 software (Mikrotek Laborsysteme GmbH, Overath, Germany).

### Hemagglutination Inhibition Test

The HI test was performed to evaluate the reactivity of ferret antiserum against 2008–09 vaccine strain A/Brisbane/59/2009, as described by the WHO manual (*17*). Antiserum was treated by receptor-destroying enzyme II (Denka Seiken, Tokyo, Japan) and adsorbed with packed turkey erythrocytes before testing to prevent nonspecific reaction. A 0.5% suspension of turkey erythrocytes was used for the HI test. Viruses with >8-fold reduced HI titer to the homologous titer of A/Brisbane/59/2009 antiserum were regarded as antigenic variants.

### Statistical Analysis

To determine the cutoff value between NAI-resistant (outlier) and -sensitive viruses, box-and-whisker plots were used. The cutoff value was defined as upper quartile + 5.0×interquartile range from the 25th to 75th percentile. In this study, ORVs with H275Y were excluded from the overall population for statistical analysis. Outliers were excluded from the calculation of mean values and standard deviations for IC_50_.

## Results

### Geographic Distribution of ORVs during the 2007–08 and 2008–09 Influenza Seasons

**Figure 2:**
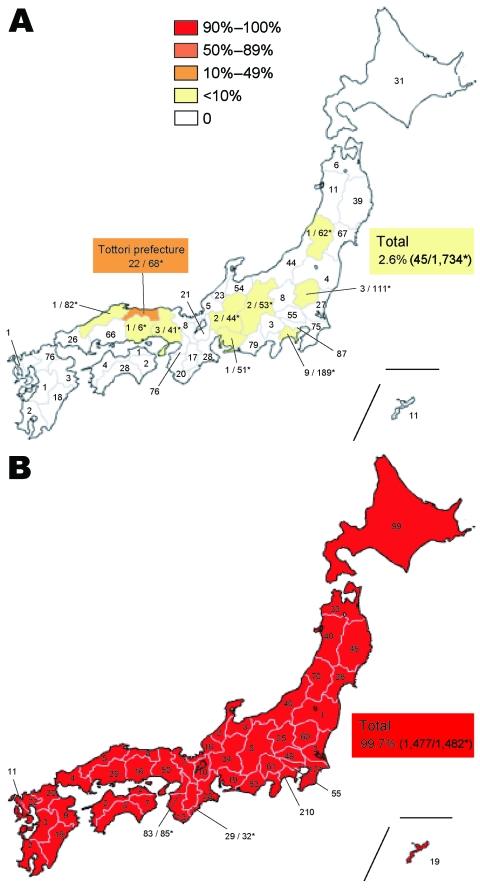
Geographic distribution of oseltamivir-resistant influenza viruses A (H1N1) (ORVs) with H275Y in Japan during the 2007–08 and 2008–09 seasons. The total number of influenza A (H1N1) isolates tested is described inside each prefecture. Total frequency in Japan was 2.6% (45/1,734) during the 2007–08 season, although a high frequency (32.4%) of ORVs was observed in Tottori prefecture (A). On the other hand, total frequency was 99.7% (1,477/1,482) during the 2008–09 season (B), indicating a drastic increase in ORVs in Japan from the 2007–08 season to the 2008–09 season. *Number of ORVs/number of subtype H1N1 isolates tested.

To estimate the frequency of influenza A (H1N1) ORVs in each prefecture of Japan, 1,734 isolates during the 2007–08 season and 1,482 isolates during the 2008–09 season were collected from all prefectures and examined by NA sequencing to detect the H275Y mutation in NA protein. In the 2007–08 season, 45 viruses possessing H275Y mutation (total frequency of ORVs 2.6%; Figure 2, panel A) were observed in 10 prefectures, indicating that the frequency of ORVs was significantly lower than that in countries in Europe and the United States (*8*,*11*–*14*). In Tottori prefecture, however, 22 of 68 influenza viruses A (H1N1) tested possessed H275Y, showing a markedly higher frequency (32.4%) than that in other prefectures. In the 2008–09 season, however, ORVs were observed nationwide. Of 1,482 influenza viruses A (H1N1), 1,477 viruses possessed a H275Y mutation, for a total frequency of 99.7% (Figure 2, panel B). These data show that ORVs increased dramatically in Japan from the 2007–08 season to the 2008–09 season.

### Genetic Analysis

Influenza viruses A (H1N1) during 2007–08 fell into either clade 2B, including the current vaccine strain A/Brisbane/59/2007, or clade 2C, and almost all influenza viruses A (H1N1) during 2008–09 fell into clade 2B. Most ORVs with H275Y belong to clade 2B, which can be further divided into 2 distinct lineages by an aspartic acid to glycine substitution at aa 354: a Northern-Eu lineage sharing 354G, which was first isolated from countries in northern Europe and now represents most ORVs worldwide; and a Hawaii lineage sharing 354D, which was first detected in Hawaii and was rarely isolated in a few countries during the 2007–08 season (Appendix Figure). In the 2007–08 season, of 45 ORVs, 1 virus (A/Yokohama/91/2007) isolated in November 2007 belonged to clade 2C, and 44 viruses fell into either the Hawaii lineage or Northern-Eu lineage. Conversely, in the 2008–09 season, all ORVs belonged to the Northern-Eu lineage, indicating that ORVs of the Northern-Eu lineage dominated in the 2008–09 season in Japan.

In the Hawaii lineage, OSVs genetically close to ORVs were observed. The NA gene of some ORVs had only 1 nucleotide difference from that of OSV counterparts (i.e., A/Tochigi/8/2008 and A/Tochigi/9/2008, A/Nagano/1100/2008 and A/Nagano/1071/2008, A/Yamagata/68/2008 and A/Yamagata/41/2008, respectively), and the other ORVs are also genetically close to OSVs from Japan (Appendix Figure). These HA genes were also genetically identical or close together (Appendix Figure), suggesting that almost all ORVs from Japan with the Hawaii lineage are derived from OSVs from Japan. On the other hand, in the Northern-Eu lineage, OSV counterparts were not observed, but foreign ORVs genetically close to ORVs from Japan were observed. During the 2007–08 season, the NA gene of ORVs from Japan was close to that of ORVs isolated from countries in Europe (i.e., A/Paris/0341/2007 and A/England/26/2008). During the 2008–09 season, the ORVs from Japan, which shared A189T on HA protein, were further divided into 4 subclades (C-1 to C-4) by common amino acid changes on HA and/or NA (Appendix Figure). ORVs from Japan in C-2 and C-3 were genetically close to the ORVs isolated from North America or Hawaii (e.g., A/Memphis/03/2008 and A/Hawaii/19/2008), whereas ORVs in C-1, representing most influenza viruses A (H1N1) from the 2008–09 season in Japan, and ORVs in C-4 were close to ORVs isolated from South Africa and Australia in the Southern Hemisphere (e.g., A/Kenya/1432/2008 and A/Victoria/501/2008). All ORVs except C-3 were isolated before the emergence of ORVs from Japan in each subclade. These findings suggest that ORVs from Japan within a Northern-Eu lineage would not have emerged domestically but instead may have been introduced from various countries.

### Antiviral Drug Susceptibility

**Figure 3:**
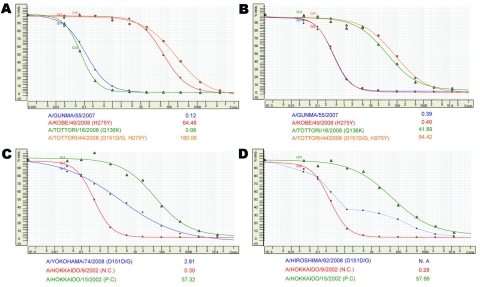
Assessment of drug concentrations required to inhibit neuraminidase activity by 50% (IC_50_) for neuramindase inhibitors (NAIs). Normal sigmoid curves were generated for most tested viruses by a neuraminidase inhibition assay for oseltamivir (A) and zanamivir (B). Sensitive A/Gunma/55/2007 (blue), oseltamivir-resistant A/Kobe/49/2008 (red) with H275Y, zanamivir-resistant A/Tottori/16/2008 (green) with Q136K, and oseltamivir/zanamivir-resistant A/Tottori/44/2008 (orange) with H275Y and D151D/G are shown. Unusual sigmoid curves were sometimes generated by the mixed population of NAI-resistant and -sensitive viruses for zanamivir: A/Yokohama/74/2008 with D151D/G (C, type A curve); and A/Hiroshima/92/2008 with D151D/G (D, type B curve). Tentative IC_50_ values (nM), shown below each panel, were obtained from type A curves but not from type B curves. NA, not available; NC, negative control; PC, positive control.

Of the 364 viruses (306 isolates in the 2007–08 season and 58 isolates in the 2008–09 season) tested by NA inhibition assay, 101 possessed a H275Y substitution. With the NA inhibition assay, although precise IC_50_ values were calculated from a normal sigmoid curve (Figure 3, panels A and B), some viruses generated 2 types of unusual sigmoid curves (Figure 3, panels C and D) resulting from the mixed population of NAI-resistant and -sensitive viruses, as previously reported (*18*). Tentative IC_50_ values were calculated from type A curves (Figure 3, panel C) and included in overall statistical analysis, but values could not be calculated from type B curves (Figure 3, panel D). Later viruses were regarded as resistant candidates.

In the NA inhibition assay for oseltamivir, OSVs showed a mean IC_50_ ± SD of 0.10 ± 0.05 nmol/L (range 0.01–0.35 nmol/L), and ORVs had a mean ± SD IC_50_ of 67.7 ± 44.1 nmol/L (range 26.1–239.2 nmol/L), showing a reduction of >260-fold in susceptibility to oseltamivir. One OSV identified as a statistical outlier (cutoff IC_50_, >0.40 nmol/L; upper quartile + 5.0× interquartile range) showed a D151E substitution on the NA protein (Table 1).

**Table 1:** Influenza virus A (H1N1) outliers to oseltamivir and/or zanamivir, Japan*

Strain	Sequence change(s)				Clinical specimen	Curve fit‡	IC_50_, nmol/L	
	D151	H275	Q136		D151		Oseltamivir	Zanamivir
Oseltamivir outlier								
A/YAMAGATA/28/2008	D/E†	H	Q		NA	Normal	0.55	0.60
Zanamivir outlier candidates								
A/TOTTORI/16/2008	D	H	K		NA	Normal	0.08	41.89
A/TOTTORI/60/2008	D	Y	Q		NA	Normal	113.86	3.64
A/KOBE/31/2008	D	Y	Q		NA	Type A	26.05	2.75
A/KOBE/32/2008	D	Y	Q		NA	Type A	135.85	3.56
A/MIE/13/2008	D/G	H	Q		D	Type A	0.18	14.80
A/YOKOHAMA/75/2007	D/G	H	Q		NA	Type A	0.13	6.53
A/HAMAMATSU/33/2008	D/G	H	Q		NA	Type A	0.13	6.15
A/TOCHIGI/30/2008	D/G	H	Q		NA	Type A	0.13	4.32
A/YOKOHAMA/74/2007	D/G	H	Q		NA	Type A	0.12	2.81
A/HIROSHIMA/92/2007	D/G	H	Q		NA	Type B	0.07	NA
A/MIE/9/2008	D/G	H	Q		D	Type B	0.08	NA
A/MIE/1/2008	D/G	H	Q		D	Type B	0.16	NA
A/MIE/14/2008	D/G	H	Q		D	Type B	0.08	NA
A/YAMAGATA/60/2008	D/G	H	Q		NA	Type B	0.19	NA
A/SAPPORO/64/2008	D/G	Y	Q		NA	Type B	147.90	NA
A/TOTTORI/44/2008	D/G	Y	Q		NA	Normal§	180.06	84.42
A/HIROSHIMA/44/2008	D/N	Y	Q		NA	Type A	239.23	2.26
A/YOKOHAMA/79/2008	D/N	Y	Q		NA	Type A	167.66	2.28
A/HIROSHIMA/46/2008	D/N	Y	Q		NA	Type A	190.35	2.40
A/HIROSHIMA/45/2008	D/N	Y	Q		NA	Type A	169.92	3.34
A/MIE/1/2009	D/N	Y	Q		NA	Type A	231.78	3.55
A/HIROSHIMA/47/2008	D/N	Y	Q		NA	Type A	106.19	4.24
A/YOKOHAMA/96/2008	D/V	Y	Q		NA	Type B	126.50	NA
Zanamivir sensitive								
A/MIE/18/2008	D/E¶	H	Q		D	Normal	0.35	1.06
A/MIE/21/2008	D/N	H	Q		D	Normal	0.22	1.18
IC_50_ mean of sensitive viruses							0.10 ± 0.05	0.40 ± 0.26
Cutoff IC_50_ values (UQ +5.0 IQR)							0.40	1.99

*IC_50_, drug concentrations required to inhibit neurominidase activity by 50%; UQ, upper quartile; IQR, interquartile range; NA, not available. Oseltamivir-resistant viruses with H275Y were excluded from overall population in statistical analysis of oseltamivir. †Mixture of D151 and D151 variants. ‡Curve fit patterns were evaluated based on Figure 3, panels C (Type A) and D (Type B). Although the viruses with D151D/G tend to generate both patterns from repeat testing for the same samples, type B was selected in this case. §Although A/TOTTORI/44/2008 showed mixed population of D151D/G, it tended to show a normal curve fit (Figure 3, panel B). ¶The IC_50_ values of most viruses with D151D/E tend to be higher than mean IC_50_ values but do not exceed the cutoff value.

In the NA inhibition assay for zanamivir, statistical analysis showed that 341 viruses were regarded as the zanamivir-sensitive viruses, with a mean ± SD IC_50_ of 0.40 ± 0.26 nmol/L (range 0.01–1.92 nmol/L), and 16 viruses (10 ORVs and 6 OSVs) were identified as outliers (cutoff IC_50_, >1.99 nmol/L) (Table 1). Seven viruses (2 ORVs and 5 OSVs) were regarded as resistant candidates from curve fit patterns. NA-sequencing for these 23 viruses (12 ORVs and 11 OSVs) showed 2 types of amino acid changes on the NA protein. One virus, A/Tottori/16/2008 (OSV), possessed a Q136K substitution, showing a high IC_50_ (41.89 nmol/L), and 19 of the other 22 viruses displayed an amino acid change G, N, or V at the D151 position (Table 1). These data suggest that D151 changes have a substantial effect on sensitivity to zanamivir (and oseltamivir). Moreover, A/Tottori/44/2008 with H275Y and D151D/G substitutions conferred high-level resistance to both NAIs (Figure 3, panels A and B). However, a recent study reported that a D151E change was detected only after virus propagation in cell culture, but not in the original clinical specimen (*19*), suggesting a possible role of cell culture in selecting these D151 variant viruses. To further investigate D151 variants, available original clinical specimens of viruses with D151 variation were subjected to NA sequencing, so that all D151 variations (D151G/E/N) were not detected in the original clinical specimens (Table 1). We thus concluded that D151 variants might not have emerged as a natural occurrence and all recent ORVs would retain sensitivity to zanamivir.

Susceptibility to M2 inhibitors was determined to find established-resistant markers by M2-sequencing. The 367 viruses (288 isolates in the 2007–08 season; 79 isolates in the 2008–09 season) including 123 ORVs (45 and 78, respectively) and 244 OSVs (243 and 1, respectively) were tested. Viruses belonging to clade 2B were sensitive to M2 inhibitors, and viruses belonging to clade 2C were resistant to M2 inhibitors, so all ORVs except A/Yokohama/91/2007 were sensitive to M2 inhibitors. A/Yokohama/91/2007 belonged to clade 2C and was the only virus resistant to both oseltamivir and M2 inhibitors.

### Antigenic Characteristics

To estimate the reactivity of ORVs and OSVs to ferret antiserum against 2008–09 vaccine strain A/Brisbane/59/2009, the HI test was performed. Good inhibition was achieved in 76% of OSVs and 69% of ORVs by A/Brisbane/59/2009 ferret antiserum, and 22% of OSVs and 28% of ORVs showed a 4-fold reduction in HI titer to the homologous titer, respectively (Table 2). Only 2% and 3% of OSVs and ORVs showed a >8-fold reduction in HI titer to A/Brisbane/59/2009 ferret antiserum. These data demonstrated that OSVs and ORVs were anitigenitically indistinguishable from each other and were similar to the 2008–09 vaccine strain A/Brisbane/59/2009.

**Table 2:** Antigenic characterization of oseltamivir-resistant and oseltamivir-sensitive influenza virus A (H1N1), Japan, 2007–2009

Antiserum	Low to homologous titer, -fold*	No. (%) sensitive, n = 169	No. (%) resistant, n = 77
A/Brisbane/59/2007	<2	128 (76)	53 (69)
	4	36 (22)	22 (28)
	>8	3 (2)	2 (3)

*Viruses with >8-fold reduced hemagglutinin inhibition titer to homologous titer were regarded as an antigenic variant.

### High Frequency of ORVs in Tottori Prefecture during the 2007–08 Season

**Figure 4:**
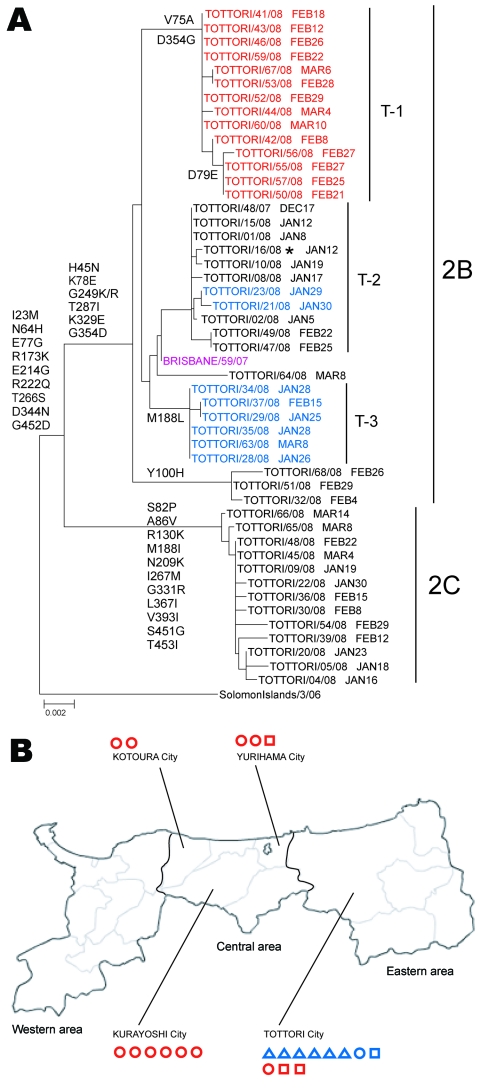
Phylogenetic analysis of influenza A (H1N1) neuraminidase genes (A) and geographic distribution of oseltamivir-resistant viruses (ORVs) (B) isolated from Tottori Prefecture, Japan, 2007–08. ORVs fell into either Northern-Eu lineage (red) or Hawaii lineage (blue); Tottori ORVs and current vaccine strains are indicated by black and purple, respectively. A) ORVs formed 3 subclades: T-1, sharing V75A and D354G; T-2, without common changes; and T-3, sharing M188L. Sampling dates are given after each strain name. Scale bar indicates nucleotide substitutions per site. B) Tottori Prefecture is geographically divided into 3 areas, comprising 19 cities. ORVs from Tottori were collected from 4 cities over 2 areas. The sampling month for each ORV is indicated by a triangle (January), circle (February), or square (March). *Zanamivir resistant.

Tottori Prefecture is located in the western part of the main island of Japan. Comprising 19 cities and geographically divided into 3 areas, this prefecture has the lowest population in Japan (Figure 4, panel B). Despite a low frequency of only 2.6% in Japan during 2007–08 season, an unexpectedly high frequency (32.4%) of ORVs was observed in Tottori prefecture (Figure 2, panel A). ORVs from Tottori were collected from 4 cities in 2 areas with no systematic bias apparent in the sampling process (Figure 4, panel B).

Phylogenetic analysis of NA genes showed that these ORVs formed 3 subclades (Figure 4, panel A): the first with a Northern-Eu lineage sharing V75A and D354G (T-1); the second with a Hawaii lineage without common changes (T-2); and the third with a Hawaii lineage and sharing M188L (T-3). Among these, only OSVs genetically close to ORVs were observed in T-2, suggesting that ORVs in T-2 would be derived from OSVs in Tottori prefecture.

A mapping study for ORVs showed that all ORVs in the Hawaii lineage were collected from Tottori city only, primarily at the end of January, whereas ORVs with the Northern-Eu lineage were collected from 4 cities, including Tottori city, during February and March. Genetically diverse ORVs belonging to T1-T3 were cocirculating only in Tottori city in the eastern area (Figure 4, panel B). The Tottori case raised concern about the possibility that these Tottori ORVs could survive to become an origin ORV for the 2008–09 season in Japan. However, phylogenetic analysis showed that all ORVs isolated during the 2008–09 season were not genetically close to ORVs from Tottori (Appendix Figure). As a result, all ORVs from Tottori seemed to have been eliminated in the 2007–08 season, and ORVs that may have been introduced from other counties were circulating during 2008–09 in Japan.

## Discussion

Our study demonstrated that ORVs dramatically increased in Japan from the 2007–08 season (2.6%) to the 2008–09 season (99.7%). All tested ORVs showed a reduction of >260-fold in susceptibility to oseltamivir by NA inhibition assay. On the other hand, almost all ORVs remained sensitive to the other antiviral-drugs, e.g., zanamivir, and M2 inhibitors. HI testing suggested that the current vaccine, A/Brisbane/59/2008, would be effective against recent ORVs. In addition, recent studies have reported that symptoms and hospitalization rates of patients infected with ORVs are no different from those seen with OSVs (*14*,*20*).

Japan has the largest per capita use of oseltamivir (>70%) in the world (*10*). Because this use could cause efficient selection of ORVs in individual patients, Japan might be the initial site of worldwide spread of ORVs. However, long-term NAI surveillance in Japan during 1996–2007 and recent surveillance showed a low frequency of NAI-resistant viruses for any strains and subtypes (*10*,*21*,*22*), suggesting that transmissibility of ORVs selected by drug pressure was remarkably decreased. In addition, previous NAI surveillance (*5*–*10*) and several animal studies (*23*–*26*) also suggested that NAI-resistant viruses would become defective viruses with attenuated infectivity and transmissibility to human. In contrast, despite little NAI use, a high emergence of ORVs has been detected in several countries in Europe since November 2007. These ORVs had as efficient transmissibility as OSVs in human-to-human transmission, resulting in worldwide spread in a short period of time. Although whether the initial ORV detected in Norway in the 2007–08 season appeared because of NAI drug pressure is unknown, those ORVs may have obtained amino acid changes on NA and/or other proteins to compensate for the defect, in addition to the H275Y substitution on the NA protein. Most ORVs belong genetically to the Northern-Eu lineage in clade 2B, suggesting that the gene constellation may contain a big advantage to retain infectivity and transmissibility.

An interesting question arose as to where the ORVs in Japan originated. In the Hawaii lineage, almost all ORVs in Japan would be derived from OSVs in Japan because the NA gene of ORVs was similar to OSV counterparts isolated at similar times or from similar regions (Appendix Figure). On the other hand, in the Northern-Eu lineage, ORVs in Japan would have been introduced from other countries. In 2007–08, almost all ORVs would be imported from countries in Europe. In 2008–09, the ORVs in C-1, which comprised most isolates in 2008–09, and ORVs in C-4 were genetically similar to ORVs isolated from the Southern Hemisphere. Because influenza activity in the Southern Hemisphere occurs half a year earlier than that in the Northern Hemisphere, most ORVs in Japan conceivably could have been imported from the Southern Hemisphere. ORVs in C-2 and C-3 were genetically similar to ORVs isolated in North America and Hawaii, but the collection month of ORVs in C-3 were similar to each other, suggesting that ORVs in C-3 might be derived from an unknown common origin ORV. The ORVs obtained during 2008–09 were not genetically similar to any ORVs isolated in Tottori during 2007–08, indicating that ORVs from Tottori had been eliminated and had not formed the origin ORVs for the 2008–09 season in Japan. As for A/Yokohama/91/2007 belonging to clade 2C, the patient from which this virus was isolated was known to have taken oseltamivir before sampling (*22*), indicating that selective drug pressure in this person might have selected for this ORV.

In the NA inhibition assay for zanamivir, some viruses, including ORVs and OSVs, showed reduced sensitivity to zanamivir. NA sequencing of these viruses showed 2 types of amino acid changes. One virus, A/Tottori/16/2008 (OSV), possessed a Q136K substitution, which reportedly confers resistance to zanamivir (*27*,*28*). Conversely, most of the other viruses possessed D151 G/V/N. The amino acid changes D151 to N or E among subtype H1N1 viruses and to A, G, E, N, or V among H3N2 have been reported (*7*,*8*,*19*), and viruses with D151 substitutions often exhibit reduced sensitivity to NAIs (*8*,*19*,*29*). However, a recent study reported a possible role for cell culture in selecting these D151 variant viruses (*19*). In the present study, D151 variations (D151G/E/N) also were not detected from available original clinical specimens (Table 1), supporting the previous finding. We thus concluded that viruses with D151 variations would not have emerged naturally, and all ORVs would remain sensitive to zanamivir.

By sequencing of M2 gene, we confirmed that almost all Japanese ORVs belonging to clade 2B retained sensitive genotype to M2 inhibitors, consistent with previously reports that recent clade 2B viruses are sensitive to M2 inhibitors, but clade 2C viruses are resistant (*27*).

During the 2007–09 seasons, we also addressed NAI surveillance for A/H3N2 and type B circulating in Japan and identified no viruses resistant to both NAIs. Conversely, in March and early April 2009, a new swine-origin influenza virus A (H1N1) (now known as pandemic [H1N1] 2009 virus) emerged in Mexico and the United States and spread rapidly to many countries, including Japan (*30*–*33*). In June 2009, detection of pandemic (H1N1) 2009 virus with H275Y on the NA protein was reported from Denmark, Hong Kong Special Administrative Region, People’s Republic of China, and Japan, but all ORVs of pandemic (H1N1) 2009 virus emerged as sporadic cases with no evidence of efficient human-to-human transmission (*34*). Although oseltamivir remains a valuable drug for treatment of pandemic (H1N1) 2009, many ORVs were isolated after prophylaxis with a half dose of the drug. Therefore, prophylaxis with oseltamivir may not be recommended as stated by WHO (*35*). Rapid and continuous monitoring of NAI-resistant viruses, including pandemic (H1N1) 2009 virus, and dissemination of the findings in timely manner remains essential.

## Supplementary Material

Appendix Figure Phylogenetic analysis of influenza A (H1N1) A) neuraminidase (NA) genes and B) hemagglutinin (HA) (HA1 region) genes. Recent influenza viruses A (H1N1) fell into either clade 2B or clade 2C. Almost all oseltamivir-resistant viruses (ORVs) with H275Y belong to clade 2B and were further divided into 2 distinct lineages: Northern-EU lineage sharing 354G (pink shading); and Hawaii lineage sharing 354D. ORVs during 2008-09 shared A189T on HA, and formed 4 subclades: C-1 (HA: G185A and S141N); C-2 (HA: G185V); C-3 (HA: G185A, NA: A86T and T339A); and C-4 (HA: G185S/N and N183S). OSVs during 2007-09, Japanese ORVs during 2007-08, Japanese ORVs during 2008-09, foreign ORVs during 2007-09 and 2008-09 current vaccine strains are indicated in black, blue, red, orange, and purple, respectively. Sampling month of each isolate is described after the strain name. Viruses resistant to zanamivir are marked with an asterisk. The phylogenetic tree of NA and HA1 genes was constructed by using neighbor-joining methods.
