# Supplementary figures and images for: Oseltamivir-Resistant Influenza Viruses A (H1N1) during 2007–2009 Influenza Seasons, Japan
Source: Emerg Infect Dis. 2010 Jun;16(6):926–35. doi: 10.3201/eid1606.091623 (PMC3086245; doi:10.3201/eid1606.091623)

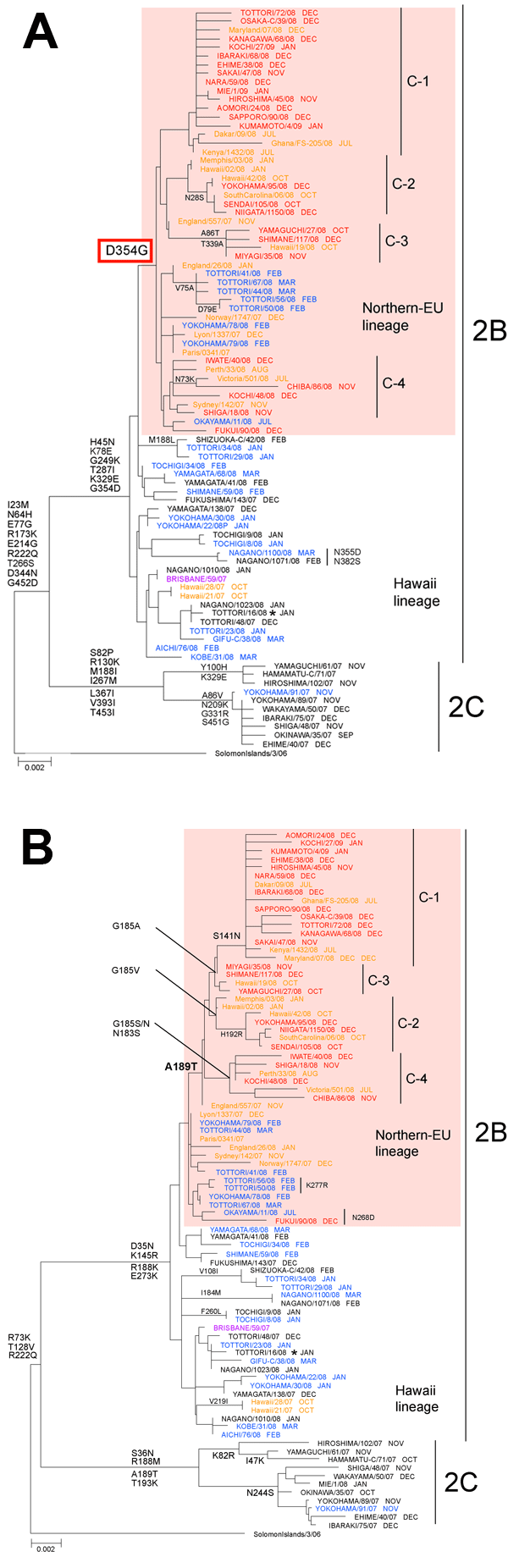

Supplement: Appendix Figure — Phylogenetic analysis of influenza A (H1N1) A) neuraminidase (NA) genes and B) hemagglutinin (HA) (HA1 region) genes. Recent influenza viruses A (H1N1) fell into either clade 2B or clade 2C. Almost all oseltamivir-resistant viruses (ORVs) with H275Y belong to clade 2B and were further divided into 2 distinct lineages: Northern-EU lineage sharing 354G (pink shading); and Hawaii lineage sharing 354D. ORVs during 2008-09 shared A189T on HA, and formed 4 subclades: C-1 (HA: G185A and S141N); C-2 (HA: G185V); C-3 (HA: G185A, NA: A86T and T339A); and C-4 (HA: G185S/N and N183S). OSVs during 2007-09, Japanese ORVs during 2007-08, Japanese ORVs during 2008-09, foreign ORVs during 2007-09 and 2008-09 current vaccine strains are indicated in black, blue, red, orange, and purple, respectively. Sampling month of each isolate is described after the strain name. Viruses resistant to zanamivir are marked with an asterisk. The phylogenetic tree of NA and HA1 genes was constructed by using neighbor-joining methods. [file 09-1623-appF-s1.gif]
